# Complement factor H supplementation rather than complete *C3* knockout provides therapeutic benefits in IgA nephropathy

**DOI:** 10.1186/s43556-026-00463-y

**Published:** 2026-04-30

**Authors:** Xianzhi Li, Xinran Ni, Xiaohan Yuan, Huan Wu, Sufang Shi, Lijun Liu, Jicheng Lv, Hong Zhang, Li Zhu

**Affiliations:** 1 Renal Division, Department of Medicine, Peking University First Hospital, Beijing, 100034 China; 2 Peking University Institute of Nephrology, Beijing, 100034 China; 3 Key Laboratory of Renal Disease (Peking University), National Health Commission, Beijing, 100034 China; 4 Key Laboratory of Chronic Kidney Disease Prevention and Treatment, Ministry of Education, Beijing, 100034 China; 5 State Key Laboratory of Vascular Homeostasis and Remodeling, Peking University and NHC Key Laboratory of Cardiovascular Molecular Biology and Regulatory Peptides, Beijing, China

**Keywords:** IgA nephropathy, Complement component 3, Complement factor H, Complement activation, Immune complex clearance

## Abstract

IgA nephropathy (IgAN), the most common primary glomerulonephritis worldwide, is characterized by mesangial IgA deposition, which triggers complement activation, primarily through the alternative pathway. While complement dysregulation contributes to kidney injury and adverse outcomes, complement may also facilitate clearance of pathogenic immune complexes. Here, we compared complete complement component 3 (C3) deficiency with supplementation of complement factor H (Cfh), the principal regulator of alternative pathway, in murine IgAN models. C3 deficiency prevented complement activation but paradoxically increased glomerular IgA deposition and macrophage infiltration, resulting in complicated inflammatory response. In contrast, Cfh supplementation reduced glomerular IgA and C3 deposition, decreased inflammatory cytokine expression, attenuated macrophage infiltration, lessened glomerular area and ameliorated proteinuria while enhancing macrophage-mediated phagocytosis of circulating IgA immune complexes. Accordingly, IgAN patients with higher plasma CFH levels exhibited reduced mesangial IgA and C3 deposition alongside elevated circulating C3 levels, supporting CFH’s protective role in regulating complement activation and promoting immune complex clearance. Our results reveal complement’s dual role in IgAN: promoting inflammatory kidney injury while facilitating clearance of pathogenic IgA deposits. Targeted complement regulation via complement factor H supplementation, rather than complete complement inhibition via global complement component 3 knockout, achieves superior therapeutic effects by simultaneously controlling complement activation, enhancing immune complex clearance, suppressing inflammation, and reducing proteinuria. These findings identify complement factor H as a novel and promising therapeutic strategy for IgA nephropathy through targeted complement regulation.

**Supplementary Information:**

The online version contains supplementary material available at 10.1186/s43556-026-00463-y.

## Introduction

IgA nephropathy (IgAN) is the most common primary glomerulonephritis worldwide [1]. Patients typically present with hematuria and varying degrees of proteinuria, and long-term outcomes are often poor, with data from UK National Registry of Rare Kidney Diseases (RaDaR) suggesting that nearly all patients are expected to progress to kidney failure within their lifetime [2]. The disease is defined by mesangial IgA deposition, frequently co-localized with complement component 3 (C3), mesangial proliferation, and inflammatory cell infiltration [3]. The presence of C3 in over 90% of patients points to a pivotal role for the complement system, which is activated primarily through the alternative pathway [4].

As a cornerstone of innate immunity, the complement system orchestrates a range of activities from direct cell lysis to the regulation of inflammation and immune clearance [5]. Its activation, which involves over 50 proteins in a cascade, converges at the cleavage of C3 [6]. This central step generates C3a, a potent anaphylatoxin, and C3b, an opsonin that drives downstream effector functions. C3b contributes to the formation of C5 convertase, leading to the production of C5a (another anaphylatoxin) and C5b, which initiates the formation of the lytic membrane attack complex [7, 8]. Concurrently, C3b and its derivatives iC3b promote phagocytosis by engaging specific receptors on immune cells, including complement receptor 1 (CR1), complement receptor 3 (CR3), complement receptor 4 (CR4) and complement receptor of the immunoglobulin superfamily (CRIg) [8]. Given these diverse functions, complement activation represents a double-edged sword, maintaining homeostasis through elimination of pathogens, damaged cells and immune complexes while simultaneously inducing potentially injurious inflammation.

This duality is particularly evident in IgAN, where the degree of complement activation strongly correlates with renal injury, inflammatory cell infiltration (including CD68^+^ cells), and clinical prognosis [9–11]. The therapeutic relevance of this pathway is underscored by recent clinical trials demonstrating that complement inhibitors can significantly reduce proteinuria in IgAN patients [12–16]. Given these findings, strategies to control complement overactivation are a major focus of research in IgAN [17]. One promising avenue, moving beyond direct inhibition of components like C3 or factor B, involves bolstering the body’s own regulatory mechanisms. This approach is supported by the established use of C1INH in angioedema [18] and by preclinical studies where engineered Cfh constructs successfully mitigated disease in murine models of C3 glomerulopathy [19–21].

In this study, we investigated two complementary strategies for modulating complement activation in a murine model of IgAN: global *C3* blockade and Cfh supplementation. Our findings aim to elucidate the complex mechanisms by which the complement system drives IgAN pathogenesis and to identify optimal therapeutic targets for its treatment.

## Results

### *C3* knockout blocks complement activation but exacerbates glomerular IgA deposition in IgAN mice

**Fig. 1:**
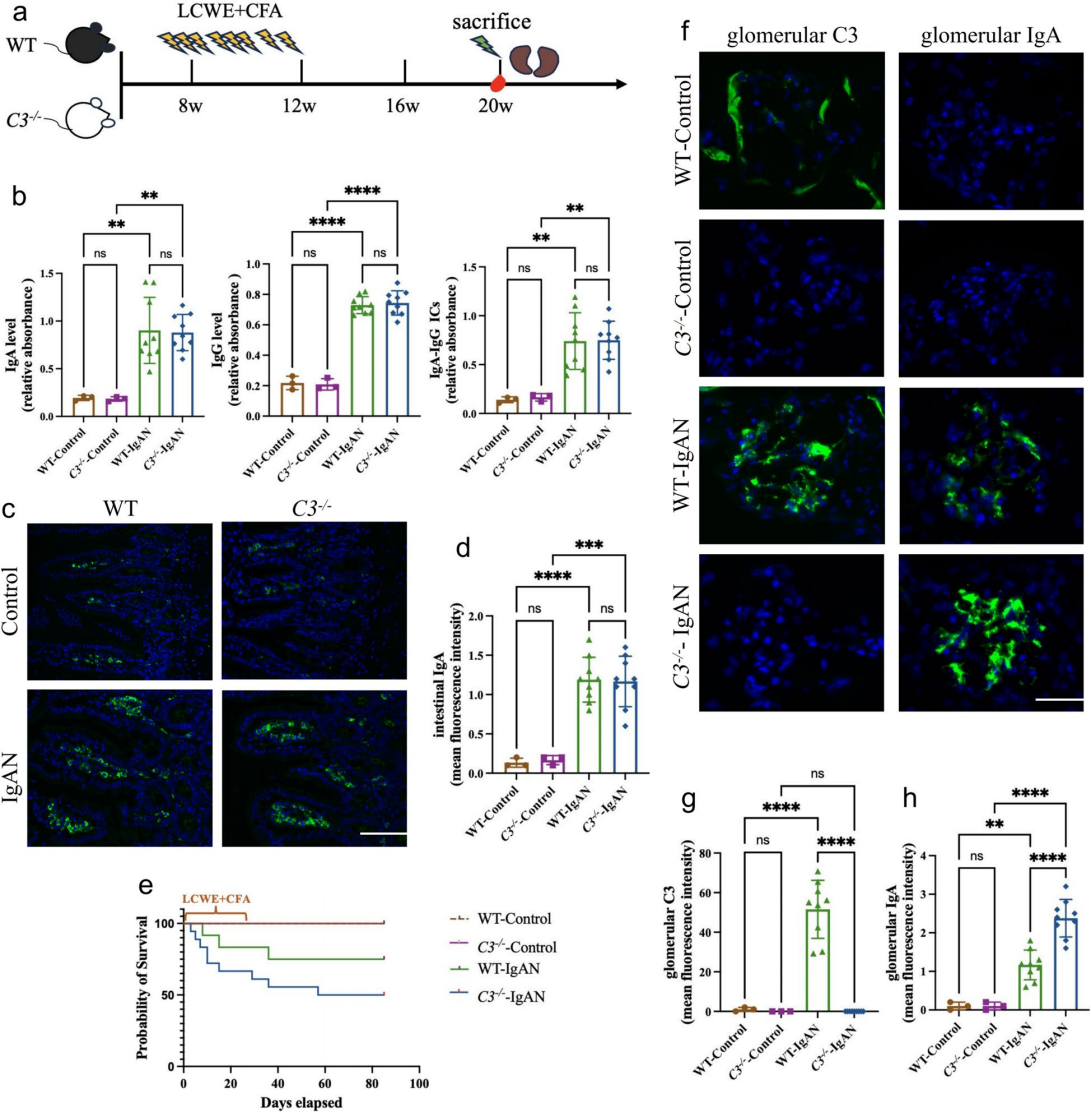
*C3* deficiency blocks complement activation but exacerbates glomerular IgA deposition in IgAN mice. **a** A schematic overview of IgAN induction in mice. 8-week-old WT and *C3*^−/−^ mice were administered intraperitoneally (i.p.) with LCWE plus CFA or PBS to induce IgAN, and observation was prolonged to 20 weeks old. **b** Circulating levels of IgA, IgG and IgA-IgG ICs in mice were detected by ELISA. **c** Representative micrographs of immunofluorescence staining of IgA in the intestinal lamina propria. magnification, × 400; scale bar, 50 µm. **d** The semiquantitative grade of IgA staining in the intestinal lamina propria. **e** Survival curves of WT and *C3*^−/−^ mice with and without IgAN induction. **f** Representative micrographs of immunofluorescence staining of IgA and C3 in glomeruli. magnification, × 400; scale bar, 50 µm. **g-h** The semiquantitative grade of C3 (**g**) and IgA (**h**) staining in glomeruli. *p ≤ 0.05, **p ≤ 0.01, ***p ≤ 0.001; ns, not significant (*p* > 0.05)

To elucidate the causal relationship between complement activation and renal pathology in IgAN, we focused on the central complement cascade component, *C3*. We utilized *C3* knockout (*C3*^−/−^) mice for our investigation. Successful ablation of *C3* expression was validated by quantifying *C3* mRNA levels in hepatic tissue and C3 protein concentrations in circulation (Fig. S1). We subsequently induced the IgAN model in both *C3*^−/−^ and wild type (WT) mice using a low-dose intraperitoneal injection regimen of CFA and LCWE to mimic early-stage IgAN (Fig. 1a).

Consistent with previous reports [22], the modeled mice demonstrated elevated levels of circulating IgA, IgG, and IgA-IgG immune complexes (IgA-IgG ICs) (Fig. 1b). Importantly, *C3* deficiency did not disrupt the IgA production system, as evidenced by comparable IgA staining in the intestinal lamina propria (Fig. 1c, d) and similar circulation IgA levels (Fig. 1b) (*p* > 0.05). Similarly, no significant differences were observed in circulating IgG and IgA-IgG ICs between *C3*^−/−^ and WT groups, irrespective of IgAN induction (Fig. 1b) (*p* > 0.05).

In the experimental IgAN models, *C3*^−/−^ mice exhibited significantly reduced survival compared to WT mice (*P* < 0.001), with survival rate of 50% versus 75%, respectively (Fig. 1e). Among surviving mice sacrificed at 12 weeks post-LCWE + CFA induction, immunofluorescence analysis of glomerular C3 and IgA revealed striking differences between groups. As expected, no C3 staining was detected in *C3*^−/−^ IgAN mice (Fig. 1f, g). However, we unexpectedly observed significantly more intense IgA deposits in *C3*^−/−^ IgAN mice compared to WT IgAN mice (Fig. 1f, h) (*p*< 0.001).

### *C3* knockout impairs macrophage-mediated glomerular IgA clearance

**Fig. 2:**
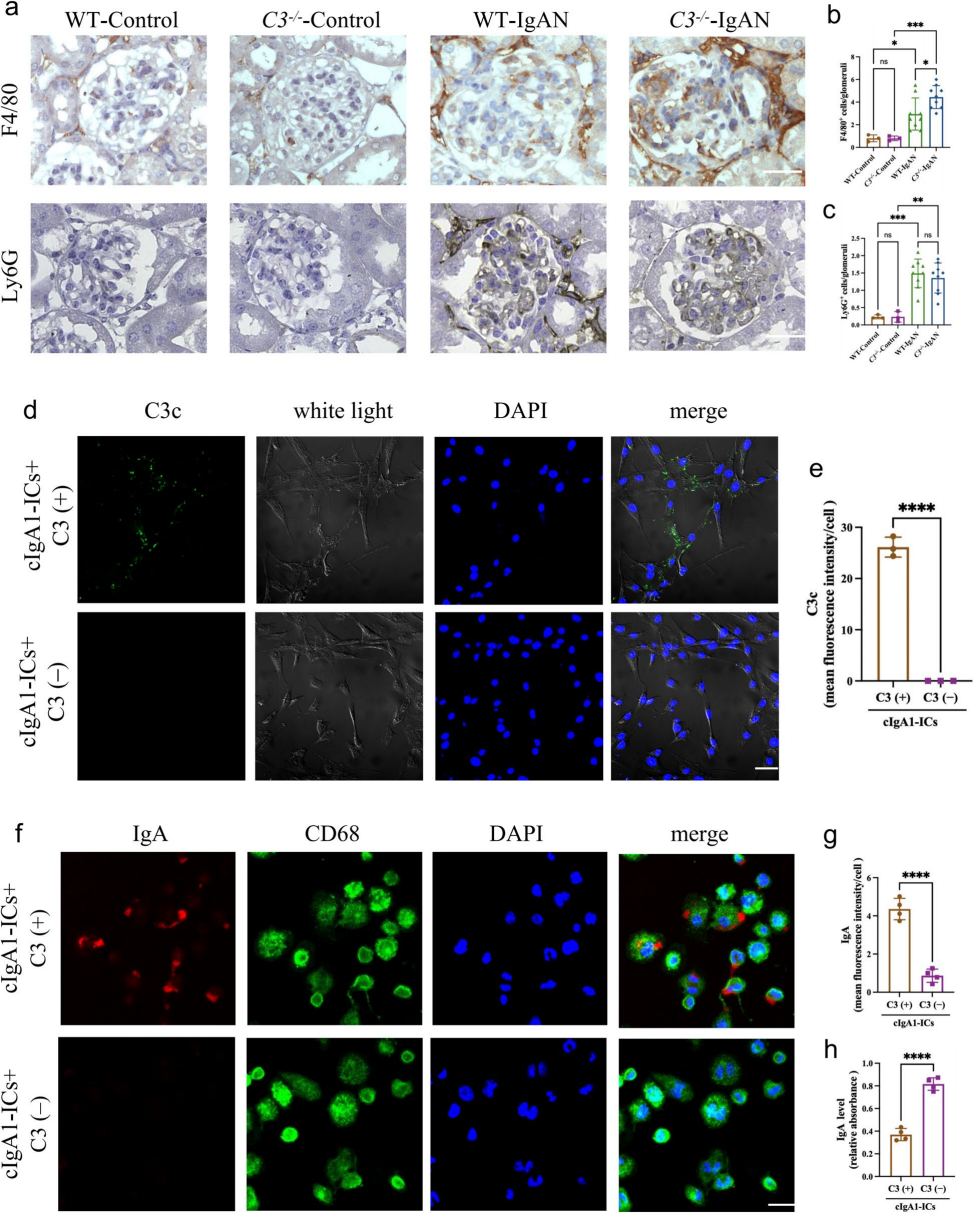
*C3* knockout impairs macrophage-mediated glomerular IgA clearance. **a** Representative micrographs of F4/80, and Ly6G staining showed glomerular macrophages and neutrophils infiltration in 20-week-old WT and *C3*^−/−^ mice. magnification, × 400; scale bar, 50 µm. **b-c** Quantitative analysis of glomerular infiltrated macrophage (**b**) and neutrophil (**c**) in mice. **d** Representative micrographs of immunofluorescence staining of C3c in human mesangial cells after cIgA1-ICs challenge. magnification, × 400; scale bar, 50 µm. **e** The semiquantitative grade of C3c immunofluorescence intensity in human mesangial cells after cIgA1-ICs challenge. **f** Representative micrographs of phagocytic cIgA1-ICs in macrophages. magnification, × 400; scale bar, 50 µm. **g** The semiquantitative grade of IgA phagocytosis by macrophages. **h** Quantification of residual IgA levels in co-culture supernatants by ELISA. *p ≤ 0.05, **p ≤ 0.01, ***p ≤ 0.001; ns, not significant (*p* > 0.05)

Macrophages and neutrophils serve as the primary phagocytes responsible for immune complex clearance during glomerulonephritis [23, 24]. Both cell types have been documented to infiltrate glomeruli of patients with IgAN [25, 26]. To investigate the roles of these phagocytes in deposited IgA clearance in *C3*^−/−^ IgAN mice, we assessed their glomerular infiltration patterns. We observed an approximately twofold increase in glomerular F4/80^+^ macrophage infiltration in *C3*^−/−^ mice compared to WT IgAN mice (*p* < 0.05) (Fig. 2a, b), while Ly6G^+^ neutrophil recruitment remained unchanged (Fig. 2a, c). This observation directed our subsequent analysis toward macrophages.

In *C3*^−/−^ IgAN mice, increased phagocyte presence coincided paradoxically with diminished clearance of deposited IgA, suggesting compromised phagocytic efficiency. To validate these findings, we stimulated primary human mesangial cells with circulating IgA1-containing immune complexes (cIgA1-ICs) in vitro. Consistent with our in vivo observations, cIgA1-ICs induced complement activation, as demonstrated by positive C3c staining on mesangial cells incubated with normal human serum, but not with C3-depleted serum (Fig. 2d, e).

We then established an in vitro phagocytosis system by co-culturing cIgA1-ICs challenged primary human mesangial cells with THP-1-derived macrophages. Compared to systems supplemented with normal serum, those containing C3-depleted serum exhibited significantly impaired phagocytic capacity for pHrodo™ labeled cIgA1-ICs (Fig. 2f, g). These complexes fluoresce upon phagocytosis in acidic intracellular environments but remain non-fluorescent at neutral extracellular pH. Quantitative analysis revealed a 78% reduction in phagocytosed IgA fluorescence intensity (Fig. 2g) and significantly elevated residual IgA levels in co-culture supernatants (Fig. 2h), confirming that C3-dependent complement activation critically enhances macrophage clearance of glomerular IgA deposits.

### *C3* deficiency partially alleviates kidney injury in IgAN mice

IgAN is characterized by a distinct immuno-inflammatory profile. Glomerular IgA deposition triggers complement activation, generation of anaphylatoxins C3a and C5a that indirectly promote local inflammation [27, 28]. Additionally, previous studies demonstrate that cIgA1-ICs directly activate mesangial cells in serum-free culture system, inducing mesangial cell proliferation and inflammatory mediator secretion [29–31]. In *C3*^−/−^ IgAN mice, despite increased IgA deposition, complement activation is blocked, creating a complex renal pathological state.

**Fig. 3:**
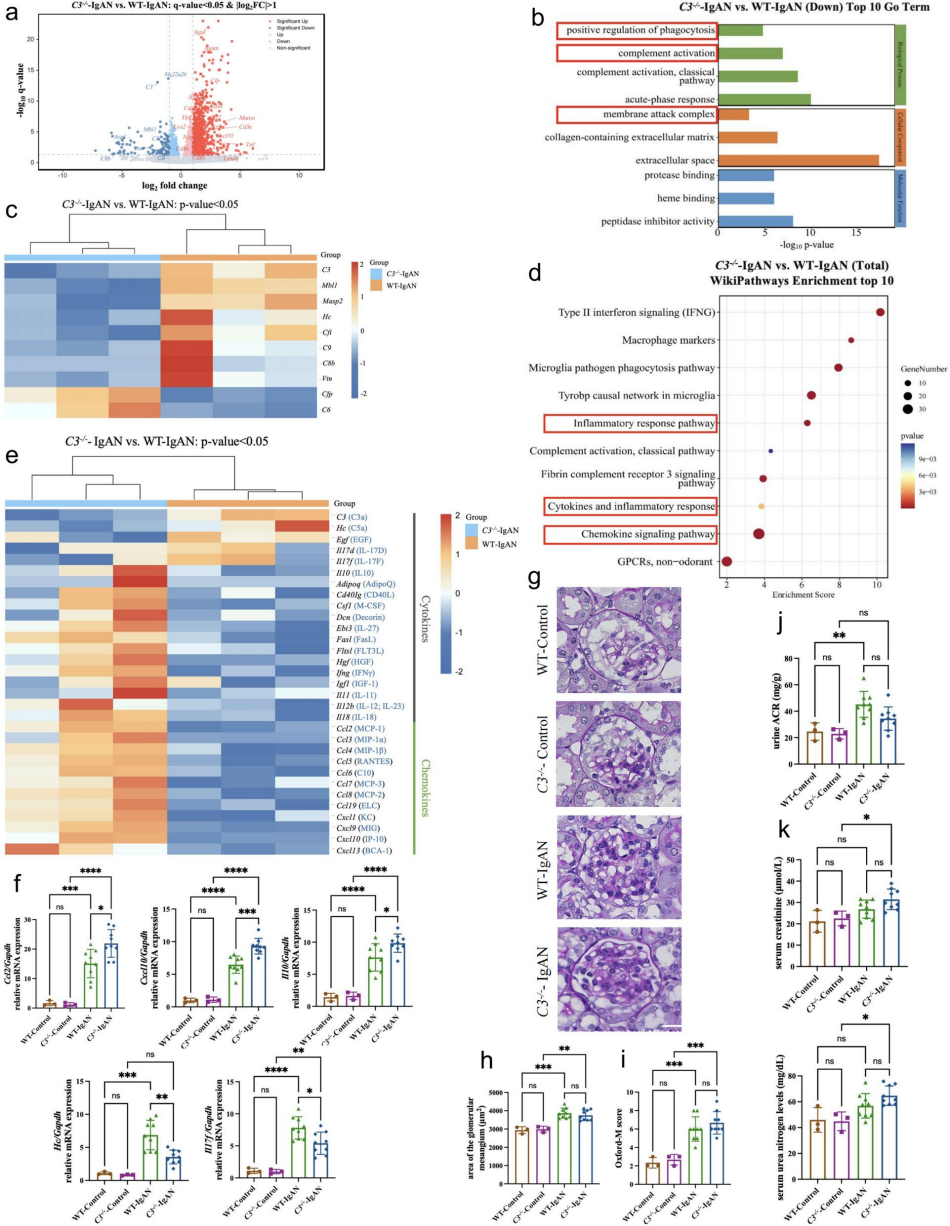
*C3* deficiency partially alleviates kidney injury in IgAN mice. **a** Volcano plot displaying differential expressed genes in the kidney of *C3*^−/−^ IgAN mice. **b** Top 10 downregulated GO enrichment pathways in kidney of *C3*^−/−^ and WT IgAN mice. **c** Heat map of differential expressed complement genes in the kidney of *C3*^−/−^ and WT IgAN mice (*p* < 0.05). **d** Top 10 Wikipathways in kidney of *C3*^−/−^ and WT IgAN mice. **e** Heat map of differentially expressed cytokine and chemokine genes in the kidney of *C3*^−/−^ and WT IgAN mice (*p* < 0.05). **f** RT-qPCR analysis of *Ccl2, Cxcl10, Il10, Hc,* and *Il17f* transcript levels of *C3*^−/−^ and WT IgAN mice kidney. **g** Representative micrographs of kidney sample PAS staining of *C3*.^−/−^ and WT IgAN mice. magnification, × 400; scale bar, 20 µm. **h** Quantitative analysis of glomerular area. **i** Semi-quantitative analysis of mesangial hypercellularity. **j-k** Quantification of urinary ACR (**j**), serum creatinine (**k**) and serum urea nitrogen levels (**k**) in IgAN mice. *p ≤ 0.05, **p ≤ 0.01, ***p ≤ 0.001; ns, not significant (*p* > 0.05)

To investigate the impact of *C3* knockout on renal injury in IgAN, we performed bulk RNA sequencing on renal tissues from *C3*^−/−^ and WT IgAN mice. Comparative analysis identified over 1,000 differentially expressed genes (DEGs; q < 0.05, |log2FC|> 1) (Fig. 3a). Gene ontology (GO) enrichment analysis revealed the suppression of complement activation and membrane attack complex (MAC) formation pathways in *C3*^−/−^ IgAN mice (*p* < 0.05) (Fig. 3b, c), confirming complement activation inhibition. Moreover, consistent with the above results, GO enrichment analysis also confirmed the downregulation of phagocytosis-related pathways in *C3* knockout mice (Fig. 3b). Wiki pathways enrichment analysis highlighted several inflammation-associated pathways affected by *C3* knockout, including inflammatory response pathway, cytokines and inflammatory response, and chemokine signaling pathway (p < 0.05) (Fig. 3d). Among the differentially expressed genes, 31 were identified as cytokines or chemokines [32], though they did not exhibit uniform directional changes. Compared to WT IgAN mice, *C3*^−/−^ IgAN mice exhibited increased expression of 26 and decreased expression of 5 pro-inflammatory factors (Fig. 3e). RT-qPCR confirmed increased *Ccl2, Cxcl10, Il10* expression alongside decreased *Hc* and *Il17f* expression (Fig. 3f). These findings indicate that in IgAN mice, both complement activation and IgA deposition contribute to renal inflammation induction, potentially through distinct pro-inflammatory cytokines pathways.

We subsequently assessed commonly used clinical indicators associated with IgAN, including proteinuria, histological lesions, and renal function. In both WT and *C3*^−/−^ mice, glomerular IgA deposition induced significant mesangial cell proliferation (Fig. 3g, h, i) and an increased urinary albumin-to-creatinine ratio (ACR) (Fig. 3j). However, serum creatinine and serum urea nitrogen levels remained unchanged (Fig. 3k) (*p*> 0.05). Notably, *C3* knock out showed a trend toward reduced urinary ACR in IgAN mice (Fig. 3j), though this improvement was not statistically significant. Regarding mesangial proliferation and renal function, *C3*^−/−^ and WT IgAN mice were comparable (Fig. 3h, i, k).

### Complement factor H supplementation attenuates both complement activation and glomerular IgA deposition in IgAN mice

**Fig. 4:**
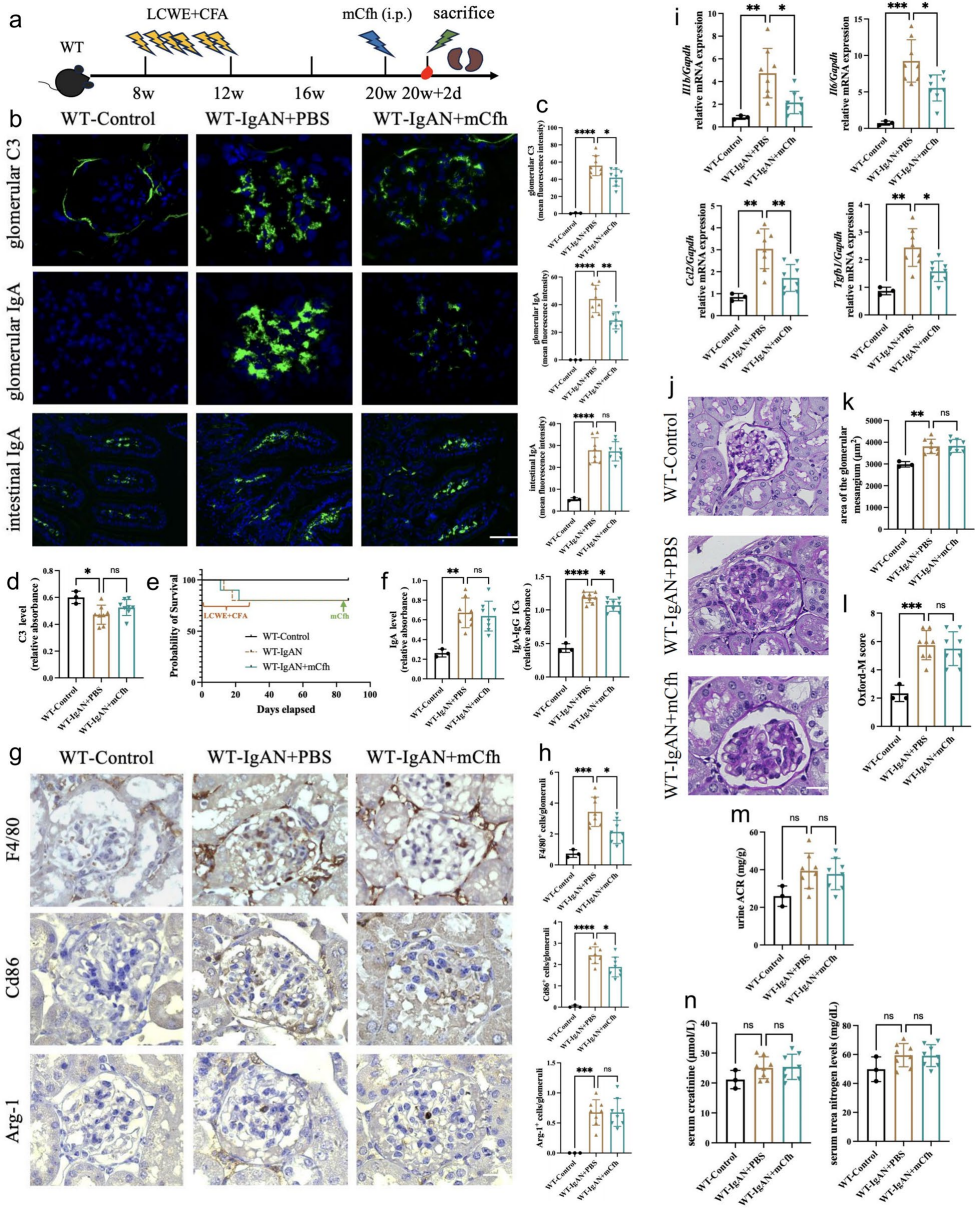
Single mCfh supplementation reduces complement activation and IgA deposition in IgAN mice. **a** A schematic overview of a single mCfh treatment to 20-week-old WT IgAN mice. Mice were subjected to mCfh intraperitoneally at a dose of 10 mg/kg in 20th week. Forty-eight hours after mCfh injection, mice were sacrificed for blood and kidney samples. **b** Representative micrographs of immunofluorescence staining of IgA and C3 in glomeruli and intestinal lamina propria. magnification, × 400; scale bar, 20 µm. **c** The semiquantitative grade of IgA and C3 staining in glomeruli and intestinal lamina propria. **d** Plasma C3 levels measured by ELISA in WT IgAN mice with or without a single mCfh treatment. **e** Survival curves of WT IgAN mice with and without single mCfh treatment. **f** Circulating levels of IgA and IgA-IgG ICs in mice were detected by ELISA. **g** Representative micrographs of immunohistochemical staining of F4/80⁺, Cd86⁺, and Arg‑1⁺ cells in glomeruli of WT‑IgAN mice treated with or without a single dose of mCfh treatment, × 400; scale bar, 20 µm. **h** Quantitative analysis of glomerular F4/80⁺, Cd86⁺, and Arg‑1⁺ cells. **i** RT-qPCR analysis of *Il1b, Il6, Ccl2,* and *Tgfb1* transcript levels in IgAN mice kidney. **j** Representative micrographs of kidney sample PAS staining of mice. magnification, × 400; scale bar, 20 µm. **k** Quantitative analysis of glomerular area. **l** Semi-quantitative analysis of mesangial hypercellularity. **m** Quantification of urinary ACR, serum creatinine (**n**) and serum urea nitrogen (**n**) levels in mice with/without a single mCfh treatment. *p ≤ 0.05, **p ≤ 0.01, ***p ≤ 0.001; ns, not significant (*p* > 0.05)

**Fig. 5:**
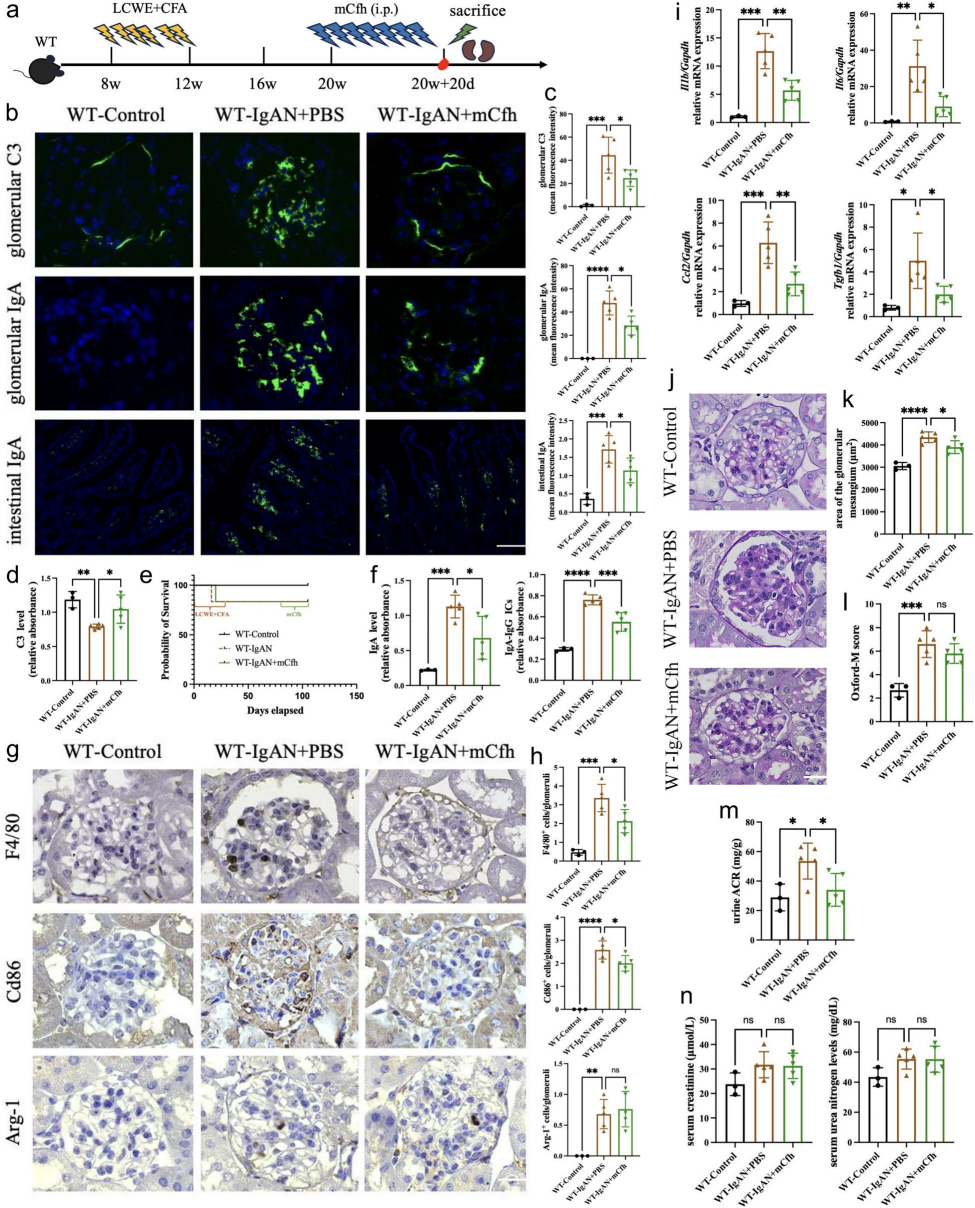
Multiple mCfh supplementations attenuate complement activation, IgA deposition, and inflammation in IgAN. **a** A schematic overview of sustained mCfh treatment to 20-week-old WT IgAN mice. Mice were subjected to mCFH intraperitoneally at a dose of 10 mg/kg every 72 h from 20th week to 23rd week. Forty-eight hours after last mCfh injection, mice were sacrificed for blood and kidney samples. **b** Representative micrographs of immunofluorescence staining of IgA and C3 in glomeruli and intestinal lamina propria. magnification, × 400; scale bar, 20 µm. **c** The semiquantitative grade of IgA and C3 staining in glomeruli and intestinal lamina propria. **d** Plasma C3 levels measured by ELISA in WT IgAN mice with or without multiple mCfh treatments. **e** Survival curves of WT IgAN mice with and without multiple mCfh treatment. **f** Circulating levels of IgA and IgA-IgG ICs in mice were detected by ELISA. **g** Representative micrographs of immunohistochemical staining of F4/80⁺, Cd86⁺, and Arg‑1⁺ cells in glomeruli of WT‑IgAN mice treated with or without multiple mCfh treatment, × 400; scale bar, 20 µm. **h** Quantitative analysis of glomerular F4/80⁺, Cd86⁺, and Arg‑1⁺ cells. **i** RT-qPCR analysis of *Il1b, Il6, Ccl2,* and *Tgfb1* transcript levels in IgAN mice kidney. **j** Representative micrographs of PAS staining of kidney sections from mice. magnification, × 400; scale bar, 20 µm. **k** Quantitative analysis of glomerular area. **l** Semi-quantitative analysis of mesangial hypercellularity. **m** Quantification of urinary ACR, serum creatinine (**n**) and serum urea nitrogen (**n**) levels in mice with/without multiple mCfh treatment. *p ≤ 0.05, **p ≤ 0.01, ***p ≤ 0.001; ns, not significant (*p* > 0.05)

These findings suggest that while complement activation induces inflammation and injury, it simultaneously facilitates deposited IgA clearance, highlighting its dual role. Therefore, complete complement activation blockade at C3 level may not represent the optimal therapeutic strategy for IgAN. A more balanced approach that modulates complement activation, reducing its intensity while preserving C3b-mediated opsonization for immune complex clearance, might yield superior outcomes. Given that complement factor H serves as the principal inhibitor of alternative pathway complement activation, which is the predominant activation pathway in IgAN, we hypothesized that complement factor H supplementation might ameliorate kidney injury caused by glomerular IgA deposition. To test this hypothesis, we recombinantly expressed and purified mouse Cfh (mCfh) protein. Following the validation of the purified mCfh’s purity, identity and complement regulatory activity (Fig. S2), we then administered this mCfh protein to WT IgAN mice via single or multiple intraperitoneal injections (Figs. 4a, 5a).

Both single and multiple mCfh treatments significantly reduced glomerular C3 deposit intensity (Figs. 4b-c, 5b-c). Concomitantly, circulating intact C3 level increased (Figs. 4d, 5d), indicating reduced complement activation. No mice deaths occurred during the mCfh supplementation period, indicating that mCfh was well-tolerated and posed no safety concerns during the intervention phase (Figs. 4e, 5e).

Initially, we hypothesized that mCfh supplementation would not increase glomerular IgA deposition due to partial preservation of complement activation and opsonization. Unexpectedly, both single and multiple mCfh treatment led to significant reductions in glomerular IgA deposit intensity in IgAN mice (Figs. 4b-c, 5b-c). Further evaluation revealed lower circulating levels of IgA and IgA-IgG ICs in treated mice (Figs. 4f, 5f). We therefore speculated that reduced circulating IgA and IgA-IgG ICs contributed to decreased glomerular IgA deposition.

Consistent with reduced glomerular IgA and C3 deposits, glomerular macrophage infiltration decreased (Figs. 4g-h, 5g-h). This reduction was primarily in CD86^+^ macrophages (M1 type; Figs. 4g-h, 5g-h), with no significant change in Arg-1^+^ (M2 type; Figs. 4g-h, 5g-h) cells, and was accompanied by reduced expression of several inflammatory factors, including *Il1b, Il6, Ccl2* and *Tgfb1* (Figs. 4i, 5i). Notably, multiple-dose mCFH treatment resulted in decreased intestinal IgA staining (Fig. 5b-c), mean glomerular area (Fig. 5j-k) and urinary ACR levels (Fig. 5m), which was not observed with single-dose treatment (Fig. 4b-c; j-k, m). However, while no significant improvements in Oxford-M score or other clinical parameters were observed with either treatment regimen (Figs. 4l, n; 5l, n).

### Complement factor H enhances macrophage phagocytosis of cIgA1-ICs

Circulating immune complex clearance is a multifaceted process. In humans, CR1 on erythrocytes plays a crucial role in transporting immune complexes to clearance sites, primarily the liver and spleen [33]. In mice, which lack CR1, immune complex transport is facilitated by CFH on platelets [34]. At hepatic and splenic sites, macrophages effectively digest and remove complexes through recognition by phagocytic receptors, including CR1, CR3, CR4 and CRIg [35–37].

Given the established roles of platelets in mouse IC transport and erythrocytes in transporting human IC transport, we at first conducted flow cytometry analysis to examine complement factor H involvement in IC transportation. We detected endogenous mCfh on mouse platelets; however exogenous mCfh did not bind to platelets (Fig. S3). In humans, we observed weak IgA signals on human erythrocytes, indicating transport of IgA-containing immune complexes by human erythrocytes. Nevertheless, human CFH (hCFH) supplementation did not alter IgA signal intensity on erythrocytes despite the presence of cIgA1-ICs in the system (Fig. S4a, b). These results suggest that complement factor H does not influence IC transportation.

**Fig. 6:**
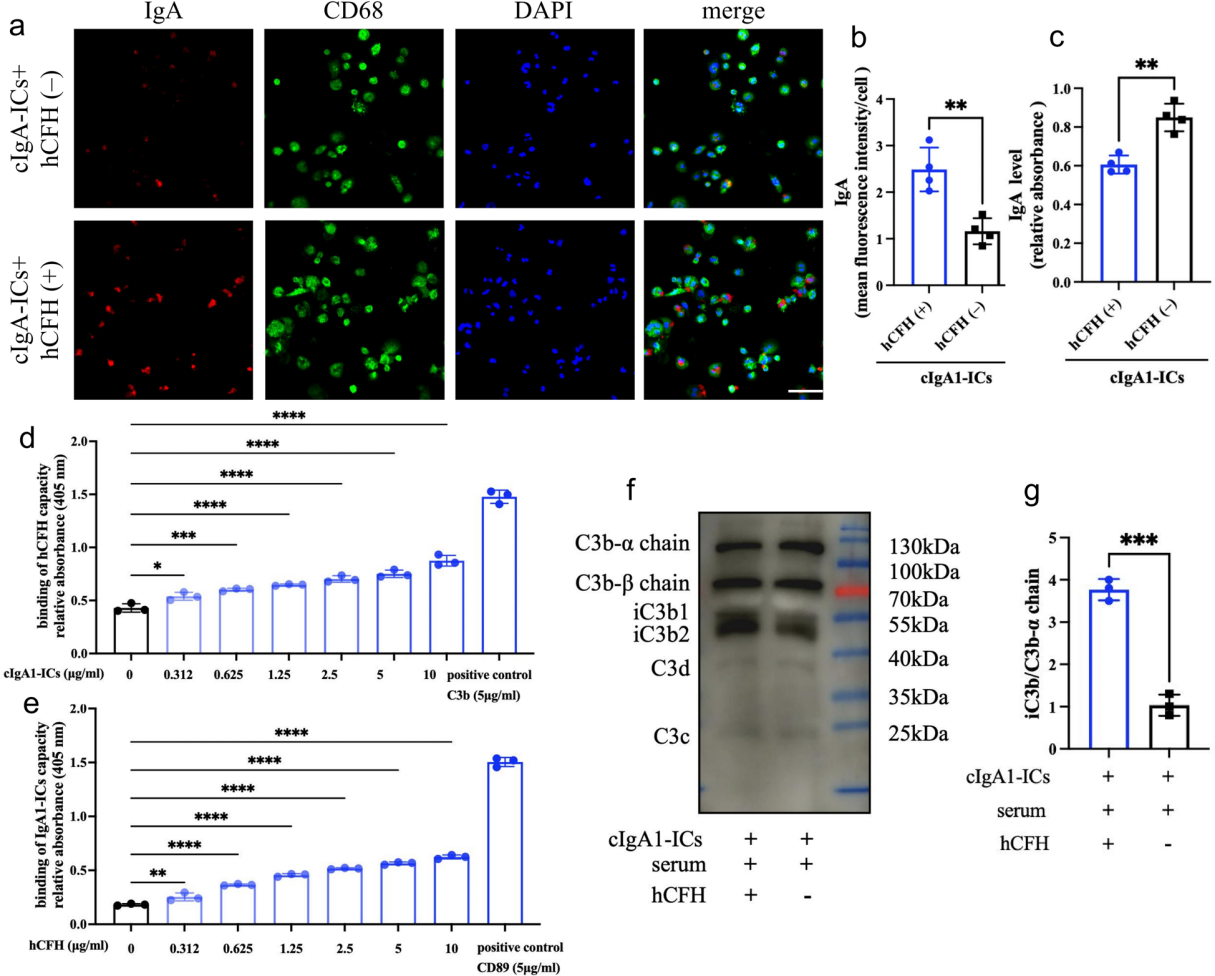
hCFH promotes macrophage phagocytosis of cIgA-ICs. **a** Representative micrographs of phagocytic cIgA1-ICs in macrophage. Images are representative. magnification, × 200; scale bar, 100 µm. **b** The semiquantitative grade of cIgA1-ICs phagocytosis by macrophages. **c** Quantification of residual IgA levels in culture supernatants by ELISA. **d** Detection of cIgA1-ICs binding to immobilized hCFH by ELISA. C3b was used as positive control. **e** Detection of hCFH binding to immobilized cIgA1-ICs by ELISA. CD89 was used as positive control. **f-g** Detection of cIgA1-ICs-bound C3 fragments by Western blot (**f**) and semi-quantitative analysis (**g**) *p ≤ 0.05, **p ≤ 0.01, ***p ≤ 0.001; ns, not significant (*p* > 0.05)

We subsequently investigated the effect of hCFH on macrophage clearance of cIgA1-ICs. Immobilized pHrodo™ labeled cIgA1-ICs were added to human macrophage culture systems with or without hCFH supplementation. Results demonstrated that exogenous hCFH promoted human macrophage phagocytosis of cIgA1-ICs (Fig. 6a, b), with correspondingly decreased cIgA1-ICs levels detected in culture supernatants (Fig. 6c). These findings suggest that hCFH promotes macrophage mediated cIgA1-ICs clearance.

To elucidate the mechanism by which hCFH reduces circulating IgA-IgG ICs, we first demonstrated direct binding between hCFH and cIgA1-ICs (Fig. 6d, e). Furthermore, compared to incubation with hCFH-depleted human serum, hCFH addition significantly enhanced the conversion of cIgA1-ICs-bound C3b into iC3b (Fig. 6f, g). Given that macrophages express multiple complement receptors involved in opsonization: CR1, which binds C3b, and CR3/CR4, which recognize iC3b, we speculated that complement factor H accelerates C3b to iC3b conversion, thereby facilitating uptake by CR3 or CR4 and subsequently enhancing macrophage phagocytosis.

### Complement factor H levels correlate with glomerular IgA deposit intensity in IgAN patients

To validate our mCfh supplementation findings from the IgAN mouse model, we analyzed a cohort of 423 patients with IgAN. Circulating CFH levels in these patients exhibited considerable variability (mean 411.15 ± 142.05 µg/mL). These levels showed no significant correlation with proteinuria, whether assessed as 24-h urinary total protein (r = −0.079, *p* = 0.116; Fig. S5a) or as the presence of nephrotic-range proteinuria (Fig. S5b). This lack of correlation argues against a substantial influence of urinary protein leakage on circulating CFH levels. We therefore divided the IgAN patients into higher and lower circulating CFH groups for subsequent analysis (mean 521.81 ± 102.11 µg/mL vs. 299.98 ± 72.72 µg/mL) (Table 1).

**Table 1:** Baseline clinical and pathological features by circulating CFH levels in IgAN

Characteristics	Total/Median (IQR)	Low CFH level group	High CFH level group	*p*-value
Number (%)	423 (100)	211 (50)	212 (50)	
CFH, μg/ml	411.15 ± 142.05	299.98 ± 72.72	521.81 ± 102.11	< 0.001
Sex (men/women, %)	199/224 (47.0/53.0)	94/117 (44.54/55.45)	105/107 (49.53/50.47)	0.331
Age, year	34.92 ± 11.67	34.40 ± 11.71	35.43 ± 11.63	0.366
24 h UTP (g/d)^a^	0.95 (0.54/1.57)	1.00 (0.54/1.74)	0.90 (0.51/1.47)	0.195
Systolic BP, mm Hg	121.99 ± 14.00	121.35 ± 13.87	122.62 ± 14.14	0.351
Diastolic BP, mm Hg	78.21 ± 11.07	78.12 ± 11.46	78.31 ± 10.70	0.862
IgA, g/L^b^	3.25 ± 1.16	3.36 ± 1.17	3.13 ± 1.14	0.076
IgG, g/L^b^	10.32 ± 2.55	10.30 ± 2.73	10.35 ± 2.33	0.866
IgM, g/L^b^	1.16 ± 0.58	1.21 ± 0.61	1.11 ± 0.54	0.110
C3, g/L^b^	1.01 ± 0.22	0.97 ± 0.20	1.06 ± 0.23	< 0.001
IgA deposition (+ to + +/+ + + to + + + +, %)	60/363 (14.2/85.8)	22/189 (10.4/89.6)	38/174 (17.9/82.1)	0.027^*^
C3 deposition (−/+ to + +/+ + + to + + + +, %)	21/206/196 (5.0/48.7/46.3)	8/93/110 (3.8/44.1/52.1)	13/113/86 (6.1/53.3/40.6)	0.048^*^
IgG deposition (−/+/+ +, %)	387/24/12 (91.5/5.7/2.8)	193/11/7 (91.5/5.2/3.3)	194/13/5 (91.5/6.1/2.4)	0.779^*^
IgM deposition (−/+/+ +/+ + +, %)	189/141/91/2 (44.7/33.3/21.5/0.5)	89/68/53/1 (42.2/32.2/25.1/0.5)	100/73/38/1 (47.2/34.4/17.9/0.5)	0.349*
Oxford classification, %				
M0/M1	96/327 (22.7/77.3)	46/165 (21.8/78.2)	50/162 (23.6/76.4)	0.728^*^
E0/E1	315/108 (74.5/25.5)	156/55 (73.9/26.1)	159/53 (75.0/25.0)	0.824^*^
S0/S1	190/233 (44.9/55.1)	85/126 (40.3/59.7)	105/107 (49.5/50.5)	0.063^*^
T0/T1/T2	249/125/49 (58.9/29.6/11.1)	118/65/28 (55.9/30.8.13.3)	131/60/21 (61.8/28.3/9.9)	0.391^*^
C0/C1/C2	176/206/41 (41.6/48.7/9.7)	87/102/22 (41.2/48.3/10.4)	89/104/19 (42.0/49.1/9.0)	0.878^*^

^*^p value for the linear-by-linear association chi-squared test

Oxford classification: mesangial hypercellularity (M), endocapillary hypercellularity (E), segmental sclerosis (S), interstitial fibrosis/tubular atrophy (T), and crescents (C)

^a^Proteinuria levels were collected from 199 patients with low CFH levels and 199 patients with high CFH levels

^b^The circulating levels of IgA, C3, IgG, and IgM were collected from 180 patients with low CFH levels and 159 patients with high CFH levels

Consistent with our observations in WT IgAN mice, IgAN patients with higher circulating CFH levels exhibited significantly reduced intensities of glomerular IgA and C3 deposits (IgA deposits, + to + +/+ + + to + + + + : 17.9%/82.1% vs. 10.4%/89.6%, *p* = 0.027; C3 deposits, −/+ to + +/+ + + to + + + + : 6.1%/53.3%/40.6% vs. 3.8%/44.1%/52.1%, *p* = 0.048) and elevated circulating C3 levels (1.06 ± 0.23 g/L vs. 0.97 ± 0.20 g/L, *p* < 0.001) compared to those with lower circulating CFH levels (Table 1).

Notably, patients with higher circulating CFH levels also showed a decreasing trend in circulating IgA levels (3.13 ± 1.14 g/L vs. 3.36 ± 1.17 g/L, *p* = 0.076), although this difference was not statistically significant. In contrast, circulating IgG (10.35 ± 2.33 g/L vs. 10.30 ± 2.72 g/L, p = 0.866) and IgM (1.11 ± 0.54 g/L vs. 1.21 ± 0.61 g/L, *p* = 0.110) levels did not differ significantly between groups. Unfortunately, in the present IgAN cohort, circulating CFH levels did not correlate with clinical manifestations or renal histopathological damage as assessed by the Oxford-MESTC classification (*p* > 0.05) (Table 1).

## Discussion

Accumulating evidence demonstrates that local complement activation in the kidneys contributes to renal injury associated with IgAN. This study provides novel insights into the roles of complement activation in IgAN, revealing that complement activation contributes to the clearance of deposited IgA immune complexes. Our findings establish the dual opposing roles of complement in IgAN and demonstrate that mCfh supplementation in IgAN mice reduces inflammation while enhancing IgA clearance, suggesting its potential as a therapeutic strategy for complement-targeted intervention in IgAN.

In IgAN, glomerular IgA deposition acts as the initial trigger of renal injury, which may subsequently activate a cascade of downstream injury mechanisms, including mesangial cell activation, inflammatory cytokine release, podocyte injury, and complement-mediated processes [27]. Given that in vivo conditions cannot isolate IgA deposition from downstream injury processes, or vice versa, it remains challenging to directly compare the renal injury effect of these factors. Multiple observational studies have demonstrated that the intensity of C3 deposition in glomeruli of IgAN patients correlates closely with Oxford classification scores and renal survival rates [11, 38], underscoring complement overactivation as one of key determinants of kidney injury in IgAN. Consequently, research has primarily focused on the detrimental effects of complement activation, leading to therapeutic interventions targeting complement proteins such as factor B (FB), C3 and C5, which have shown significant inhibition of complement activation and reduction in proteinuria.

To explore the mechanism of complement inhibition in IgAN, we employed an acute/subacute IgAN mouse model [22]. This model, which primarily mimics early disease drivers such as elevated circulating IgA and glomerular IgA deposition, is induced by peritoneal injection of LCWE plus CFA. It is not designed to model chronic progressive kidney injury, and accordingly, the renal pathology is generally mild. Most histological lesions are confined to mesangial hypercellularity (Oxford M lesion), with other chronic lesions being rare. In this model, *C3* knockout effectively blocked complement activation, as evidenced by the complete absence of glomerular C3 staining and the suppression of complement activation and membrane attack complex (MAC) formation pathways in bulk RNA sequencing of renal tissues. Unexpectedly, completely *C3* deficiency through *C3* knockout significantly increased glomerular IgA deposition intensity. These complex effects of complement on glomerular immune complex processing have been reported previously [39, 40]. Studies involving *C4* and *C3* knockout mice showed reduced glomerular IgG deposition but increased IgM and IgA deposition following apoferritin immunization [41]. Additionally, factor D-deficient mice spontaneously developed mesangial immune complex glomerulonephritis with associated albuminuria and impaired renal function [42], suggesting that complement activation might take part in deposited IgA clearance through its opsonization function.

Our in vitro mesangial cell-macrophage coculture system directly demonstrates that local complement activation enhances macrophage-mediated phagocytosis of deposited IgA. While excessive complement activation promotes glomerular inflammation in IgAN [43], our findings suggest that C3 facilitates IgA clearance through mechanisms distinct from its proinflammatory effects. This dichotomy is evident in *C3*^−/−^ IgAN mice, where absent C3 deposition correlates with both the inhibition of complement activation and exacerbated IgA accumulation. This complex phenotype was further reflected in the expression of pro-inflammatory factors, which showed bidirectional changes. On one hand, certain pro-inflammatory factors (including *Ccl2* and *Cxcl10*) were increased, likely due to mesangial cells activation by pathogenic IgA1-containing immune complexes [29–31]. The elevated *Ccl2* expression, encoding Mcp-1, contributed to macrophage infiltration in *C3*^−/−^ IgAN mice, further increasing pro-inflammatory factor production. On the other hand, other factors, such as *Hc* and *Il17f*, were downregulated, possibly as a consequence of complement blockade. Consistent with these findings, we observed no significant improvement in renal injury in *C3*^−/−^ IgAN mice, except for a trend toward reduced urinary ACR. This outcome likely reflects a balance between the injury-promoting effects of enhanced IgA deposition and the injury-limiting effects of complement suppression. This further supports the notion that both IgA deposition and complement activation contribute to renal injury in IgAN. Notably, the lack of significant renal protection in *C3*^−/−^ mice appeared to differ from the proteinuria reduction achieved with pharmacological C3 inhibition (ARO-C3). This discrepancy likely stems from fundamental differences between the two interventions: *C3*^−/−^ IgAN mice model a complete, systemic and lifelong deficiency, whereas pharmacological inhibition is partial, transient and initiated in adulthood.

These findings support a model wherein C3 functions as a molecular rheostat in IgAN: its activation drives inflammatory cascades (likely through C3a) while maintaining homeostatic immune complex clearance via C3b opsonization-dependent phagocytic pathways. This mechanistic dichotomy highlights the need for refined therapeutic strategies that selectively modulate rather than completely block complement activation, preserving physiological immune complex clearance while suppressing pathological inflammation.

Complement activation in IgAN occurs primarily through the alternative pathway, with complement factor H serving as a natural inhibitory regulator and identified as an IgAN susceptible gene by GWAS [44, 45]. Our IgAN mouse model experiments reveal that transient mCfh administration reduces IgA and C3 deposition, as well as macrophage infiltration without immediately affecting renal phenotype, while moderate-duration mCfh supplementation significantly decreases renal IgA deposition and attenuates proteinuria. These findings highlight complement factor H as a central regulator in restoring immune balance: mitigating local glomerular complement activation to reduce renal injury while preserving sufficient C3 activation to facilitate glomerular IgA clearance.

An important unexpected finding is that prolonged mCfh supplementation decreased circulating levels of IgA and IgA-IgG ICs in IgAN mice, suggesting mCfh involvement in systemic IgA metabolism. Mechanistically, we found that cIgA1-ICs induced complement activation with C3b binding, which was in accordance with previous report [46]. Meanwhile, we found that hCFH accelerated C3b degradation to iC3b through its cofactor activity. C3b and iC3b are recognized by different phagocyte receptors, CR1 and CR3/CR4, respectively. In neutrophils, CR1 primarily promotes particle adhesion while CR3 mediates the subsequent engulfment [47]. Therefore, we hypothesize that hCFH-induced C3b to iC3b conversion enhances cIgA1-ICs clearance by phagocytes. This was validated by observed enhanced macrophage phagocytosis of cIgA1-ICs with hCFH addition. Besides that, the decreased circulating IgA levels following multiple hCFH supplementation aligned with reduced IgA staining in intestinal lamina propria (one of the main sites for IgA production), suggesting complement factor H may influence circulating IgA levels partly through reduced intestinal IgA production, although the exact mechanism requires further exploration.

Additionally, we demonstrate that circulating CFH levels inversely correlate with glomerular IgA and C3 deposition in our IgAN cohort, consistent with our observations in CFH supplemented IgAN mice. However, unlike in mice, CFH levels were not associated with proteinuria in patients, with comparable proteinuria between high and low CFH groups. A potential explanation for this discrepancy lies in the widespread use of ACEi/ARB therapy in patients, which reduces proteinuria through hemodynamic effects and may have masked any CFH-related benefit. The lack of detailed treatment records from the months prior to enrollment, precluded us from adjusting for confounding by indication and limited our ability to detect a potential independent association between CFH and proteinuria in this clinical setting.

Our study had several limitations. First, the association between circulating CFH levels and glomerular IgA deposition was observed only in our single-center cohort. While a European IgAN cohort showed that *CFHR3-1Δ* (a genetic variant with increased circulating CFH levels) associated with higher glomerular IgA and IgG deposits [48], validation in independent IgAN cohorts is needed. Additionally, the potential causal effect of complement factor H on proteinuria remains to be elucidated in comprehensively documented IgAN patient cohorts. Second, the IgAN mouse model utilized in this study was an acute/subacute model with only mild renal pathology and preserved renal function. This precluded a thorough investigation into the effects of sustained glomerular IgA deposition and complement activation on the progression of kidney injury. Furthermore, the characteristics of the cross-sectional observational study in the IgAN cohort limit our ability to explore the causal relationship between CFH and renal phenotypes in patients with IgAN. Additionally, the absence of follow-up data in our IgAN patients prevented determination of whether circulating CFH levels can predict IgAN progression. Third, although we demonstrated that completely complement blockade impairs IgA clearance, the effect of pharmacological C3 inhibitors, which typically result in partial, reversible inhibition, may differ from the complete blockade used in our study and requires further evaluation. Regarding complement regulatory therapies in IgAN, our study focused on CFH supplementation. However, other promising strategies, such as CFI supplementation or the use of soluble CR1, warrant future investigation. Moreover, the optimal extent of complement modulation and CFH supplementation regime remain to be determined. Dedicated pharmacokinetic and pharmacodynamic studies are needed to optimize the molecular form (full-length CFH versus engineered variants), dosage, frequency, and route of administration to maximize therapeutic benefit in IgAN. Fourth, while our study investigated glomerular IgA deposition and complement activation, multiple other factors and mechanisms contribute to kidney injury in IgA nephropathy. Our present study was unable to compare the relative contributions of these factors, which continues to represent an important objective for future research in the field.

In conclusion, our study identifies dual opposing roles of complement activation in IgAN: inducing inflammation that causes kidney injury while facilitating clearance of glomerular IgA-containing immune complexes. Complete complement blockade exacerbates glomerular IgA deposition and macrophage infiltration. Complement modulation through complement factor H supplementation demonstrates therapeutic efficacy by concurrently reducing IgA deposition, complement activation, inflammation, and proteinuria. These findings suggest complement factor H supplementation as a promising complement-targeted therapeutic strategy for IgAN. Further research is required to translate this therapeutic concept into clinical applications for patients with IgAN.

## Methods

### Study population

A total of 423 patients diagnosed with IgAN at Peking University First Hospital between 2008 and 2013 were enrolled in this study. The diagnosis of IgAN was established by renal biopsy, characterized by predominant mesangial IgA deposition on immunofluorescence and confirmed by the presence of mesangial electron-dense deposits on electron microscopy. Patients with secondary IgAN, IgA vasculitis, or ANCA-associated vasculitis were excluded following a comprehensive evaluation. Peripheral venous blood samples were collected at the time of renal biopsy in EDTA-anticoagulated tubes, centrifuged at 3,000 rpm for 15 min at 4 °C, and plasma aliquots were stored at −80 °C until CFH quantification by sandwich ELISA, as previously described [49]. Clinical and pathological data including circulating complement C3, immunoglobulin levels (IgA, IgG, IgM), proteinuria, and semiquantitative assessments of glomerular mesangial immune deposits were obtained from biopsy-era medical records. The study was approved by the Ethics Committee of Peking University First Hospital (approval No. 2025R0547), and written informed consent was obtained from all participants.

### Antibodies and reagents

Primary antibodies included: Alexa Fluor 488-conjugated anti-mouse IgA, IgM, and IgG (SouthernBiotech, 1040–30, 1021–30, 1030–30); FITC-conjugated anti-mouse C3 (MP Biomedicals, 0855500); anti-mouse C3 (HycultBiotech, HM1045); HRP-conjugated anti-mouse immunoglobulins and C3 (SouthernBiotech and MP Biomedicals); anti-mouse Ly6G (Abcam, ab238132), F4/80 (Cell Signaling Technology, 70076), Cd86 (Cell Signaling Technology, 10589), and Arginase-1 (Cell Signaling Technology, 93668); anti-human CFH antibodies (Thermo, MA5-17735; Quidel, A312; Merck, 341276); and HRP-conjugated anti-human IgA (Abcam, ab98558). Secondary antibodies included Alexa Fluor 488-conjugated goat anti-mouse and anti-rabbit IgG, HRP-conjugated anti-rabbit and anti-goat IgG, and FITC-conjugated avidin.

cIgA1-ICs were purified from IgAN patient plasma using Jacalin affinity chromatography followed by Sephacryl S-300 gel filtration, as previously described [50]. Human CFH was purchased from Complement Tech (Catalog#: A137). Recombinant mouse Cfh protein was expressed in HEK-293F cells using established protocols [50]. Briefly, the full-length mouse *Cfh* cDNA was cloned into a mammalian expression vector with a C-terminal 6xHis tag. The plasmid was transfected into HEK293 cells using polyethylenimine (PEI). The protein was secreted into the culture supernatant, which was harvested after 4 days. mCfh was initially purified by immobilized metal affinity chromatography (IMAC) using Ni–NTA resin, followed by buffer exchange and concentration into PBS using centrifugal filtration. The purity, identity and complement regulatory activity of the purified mCfh protein were verified by Coomassie Brilliant Blue staining, Western blotting and hemolysis assay respectively. Detailed experimental protocols are described in the Supplemental Methods.

### Animal studies

The *C3* knockout mice were a generous gift from Professor Yi Wu (Xi’an Jiaotong University) [51]. Wild-type (WT) mice on C57BL/6 J background were obtained from the Jiangsu Jicui Laboratory Animal Science and Technology Co., Ltd. *C3*^⁻/⁻^ and WT mice were maintained under specific pathogen-free conditions with ad libitum access to food and water. All procedures were approved by the Laboratory Animal Care and Use Committee of Peking University First Hospital (approval No. J2024096).

IgAN was induced in 8-week-old male mice through repeated intraperitoneal injections of Lactobacillus casei cell wall extract (LCWE) emulsified in complete Freund’s adjuvant, following established protocols [22]. Mice were euthanized 12 weeks post-initial treatment for endpoint analyses.

In line with previous reports on mini factor H supplementation in mice, we employed a similar molar concentration of mCfh [52, 53]. For mCfh treatment studies, WT IgAN mice received either single-dose recombinant mCfh (10 mg/kg intraperitoneally, 48 h before euthanasia) or multiple doses (10 mg/kg every 72 h for 7 doses, starting 12 weeks post-LCWE induction). Age-matched controls received equivalent volumes of PBS.

Glomerular deposits and inflammatory cell infiltration were evaluated through conventional immunofluorescence and immunohistochemical staining techniques. Biochemical parameters were assessed using commercially available ELISA kits, including urinary albumin and creatinine, as well as blood creatinine and urea nitrogen concentrations. Detailed experimental protocols are described in the Supplemental Methods.

### RNA sequencing and bioinformatics

Total RNA extraction was performed using TRIzol reagent (Invitrogen). Library preparation utilized the VAHTS Universal V6 RNA-seq Kit, with sequencing conducted on the Illumina NovaSeq 6000 platform (150 bp paired-end reads) by OE Biotech Co., Ltd. (Shanghai, China).

Quality control was performed using fastp (S4), followed by genome alignment with HISAT2. Gene expression was quantified as FPKM values, with read counts obtained using HTSeq-count. Differential expression analysis employed DESeq2, while hierarchical clustering and pathway enrichment analyses were conducted using R (v3.2.0) and ClusterProfiler packages, respectively.

### Cell culture experiments

cIgA1-ICs were fluorescently labeled using pHrodo™ iFL RED STP ester (Thermo Fisher Scientific) and incubated at 150 μg/ml with or without recombinant hCFH (350 μg/ml). THP-1 monocytes (Procell) were cultured in RPMI-1640 supplemented with 10% FBS, 0.05 mM β-mercaptoethanol, and 1% penicillin/streptomycin at 37 °C under 5% CO₂. Macrophage differentiation was induced using 100 nM phorbol 12-myristate 13-acetate (PMA) for 48 h in 8-well chamber slides.

For phagocytosis assays, differentiated macrophages were incubated with labeled cIgA1-ICs and 10% human serum, with or without hCFH for 4 h at 37 °C. Supernatants were collected for IgA quantification by ELISA, while cells were fixed in 4% paraformaldehyde for CD68 immunostaining and fluorescence microscopy (Leica).

Primary human mesangial cells (HMC; ScienCell) were cultured in specialized medium containing 2% FBS and 1% growth supplement. For coculture experiments, HMCs and THP-1 macrophages were seeded in direct contact and treated with pHrodo™-cIgA1-ICs in the presence of either C3-depleted serum or normal human serum for 4 h.

### Enzyme-linked immunosorbent assays

Serum immunoglobulin levels were measured using sandwich ELISA with overnight coating of goat F(ab’)₂ anti-mouse Ig, followed by 1% BSA blocking and detection with HRP-conjugated specific antibodies. Circulating IgA-IgG ICs were quantified via cross-capture ELISA using anti-IgA capture and anti-IgG detection antibodies. Similarly, circulating C3 levels in mice were measured using ELISA. Plates were coated overnight with rat anti-mouse C3 monoclonal antibody, followed by blocking and washing. Diluted mouse plasma samples were then added, and bound C3 was detected using HRP-conjugated goat anti-mouse C3 antibody.

hCFH-cIgA1-ICs binding interactions were assessed using serial dilutions (0–10 μg/ml) with reciprocal binding partners detected by HRP-conjugated antibodies. All reactions were developed with TMB substrate, stopped with 1 M H₂SO₄, and analyzed at 450 nm using a Bio-Rad Model 550 microplate reader.

### Quantitative real-time PCR

Total RNA was extracted using TRIzol reagent, with reverse transcription performed using the FastKing One-Step RT Kit (Tiangen Biotech). Quantitative PCR employed SYBR Prime Script RT-PCR kit (Applied Biosystems) with gene expression calculated using the 2^−ΔΔCt^ method normalized to GAPDH. Primer sequences are provided in Table S1.

### ELISA-elution-Western blot assay

The mechanism of C3b conversion on cIgA1-IC surfaces was validated using CFH-deficient serum by an ELISA-Elution-Western blot assay. Plates were coated with cIgA1-ICs diluted in carbonate buffer overnight at 4 °C. After blocking with 3% BSA-PBST, wells were incubated with normal human serum (NHS) or CFH-deficient serum for 1 h at 37 °C to allow complement activation and C3 deposition. Following extensive washing, bound proteins were eluted in 5 × SDS loading buffer containing β-mercaptoethanol at 95 °C. The eluates were collected and analyzed by Western blotting. Briefly, samples were subjected to SDS-PAGE, transferred onto PVDF membranes, and immunoblotted with anti-human C3c antibody. Protein bands were visualized using a chemiluminescent substrate and detected with an imaging system. The ratio of iC3b to C3b was assessed to evaluate cleavage efficiency.

### Statistical analysis

Continuous variables with normal distribution are presented as mean ± standard deviation and analyzed using Student’s t-test (two groups) or one-way ANOVA (≥ 3 groups). Categorical variables were compared using chi-square tests. Statistical analyses were performed using SPSS 27.0, with graphs generated in GraphPad Prism 9.0. Statistical significance was set at *p*≤ 0.05 (two-tailed).

## Supplementary Information

Supplementary Material 1.

## Data Availability

The raw sequence data reported in this paper have been deposited in the Genome Sequence Archive (Genomics, Proteomics & Bioinformatics 2025) in National Genomics Data Center (Nucleic Acids Res 2025), China National Center for Bioinformation (GSA: CRA041499) that are publicly accessible at https://ngdc.cncb.ac.cn/gsa. Raw data are available for reanalysis upon reasonable request to the corresponding author.
