# Supplementary material for: Complement factor H supplementation rather than complete C3 knockout provides therapeutic benefits in IgA nephropathy
Source: Mol Biomed. 2026 Apr 30;7:61. doi: 10.1186/s43556-026-00463-y (PMC13133335; doi:10.1186/s43556-026-00463-y)

**Title:** Complement factor H supplementation rather than complete *C3* knockout provides therapeutic benefits in IgA nephropathy

**Authors:** Xianzhi Li^1,2,^ Xinran Ni^1,2^, Xiaohan Yuan^1,2^, Huan Wu^1,2^, Sufang Shi^1,2^, Lijun Liu^1,2^, Jicheng Lv^1,2^, Hong Zhang^1,2^, Li Zhu^1,2^

**Affiliations:** ^1^ Renal Division, Department of Medicine, Peking University First Hospital; Peking University Institute of Nephrology; Key Laboratory of Renal Disease (Peking University), National Health Commission; Key Laboratory of Chronic Kidney Disease Prevention and Treatment, Ministry of Education, Beijing 100034, China.

^2^ State Key Laboratory of Vascular Homeostasis and Remodeling, Peking University and NHC Key Laboratory of Cardiovascular Molecular Biology and Regulatory Peptides.

**Corresponding author:**

Li Zhu

Email: funnyzhuli@bjmu.edu.cn

**Supplementary Materials**

Index

[Supplemental Methods 2](#_Toc223821941)

[Tables 4](#_Toc223821942)

[Supplemental Table S1. RT-qPCR primer sequences. 4](#_Toc223821943)

[Figures 5](#_Toc223821944)

[Supplementary Figure S1. Validation of *C3* knock out in *C3*^-/-^ mice. 5](#_Toc223821945)

[Supplementary Figure S2. Validation of recombinant mouse Cfh protein. 6](#_Toc223821946)

[Supplementary Figure S3. Detection of Cfh on mouse platelets. 7](#_Toc223821947)

[Supplementary Figure S4. Detection of IgA containing immune complexes on human erythrocytes. 8](#_Toc223821948)

[Supplementary Figure S5. Plasma CFH levels in IgAN patients stratified by severity of proteinuria. 9](#_Toc223821949)

# **Supplemental Methods**

***Validation of recombinant expressed mCfh protein***

The purified mCfh protein was subjected to SDS-PAGE, followed by Coomassie Brilliant Blue staining to assess its purity. For identity confirmation, Western blotting was performed using a goat anti-human CFH antibody, which cross-reacts with and specifically recognizes the purified protein.

The complement regulatory function of purified mCfh was evaluated using a hemolysis assay under conditions favoring alternative pathway activation. Washed rabbit erythrocytes (1 × 10⁷ cells) were incubated with 10% CFH-depleted normal human serum (Quidel) in alternative pathway (AP) buffer (0.15 mM CaCl₂, 141 mM NaCl, 5.5 mM MgCl₂, 1.8 mM sodium barbital, 3.1 mM barbituric acid, 0.1% gelatin, and 5 mM EGTA, pH 7.0) in the absence (control, defined as 100% hemolysis) or presence of purified mCFH at final concentrations of 10 μg/mL or 100 μg/mL. Baseline control wells contained 40 mM EDTA to completely inhibit hemolysis. After incubation at 37°C for 40 min, the reaction was terminated by adding 40 mM EDTA buffer, and the absorbance of the supernatant was measured at 405 nm. The percentage of hemolysis for each sample was calculated as: Hemolysis (%) = [(OD_sample_ – OD_EDTA_) / (OD_control_ – OD_EDTA_)] × 100%, where OD_control_ is the absorbance of the control sample (without added CFH) and OD_EDTA_ is the absorbance of the baseline control (with EDTA).

***Histological and immunological analyses***

Renal tissues were fixed in 4% paraformaldehyde, paraffin-embedded, and sectioned at 2 μm thickness. Periodic acid-Schiff staining was performed for morphological assessment, with mesangial hypercellularity quantified as cells per mesangial area. Furthermore, to more accurately assess mesangial proliferation and glomerular injury, we calculated the mean glomerular area from 25 cortical glomeruli per kidney using ImageViewer software.

Immunohistochemical staining for F4/80, Ly6G, Cd86, and Arg-1 employed heat-mediated antigen retrieval in Tris-EDTA buffer (pH 9.0) followed by primary antibody incubation (anti-F4/80, 1:400; anti-Ly6G, 1:1000; anti-Cd86, 1:200; anti-Arg-1, 1:400) and HRP-conjugated secondary detection with DAB chromogen. Glomerular infiltration was quantified by counting positive cells across 20 randomly selected glomeruli per animal.

Immunofluorescence analysis of immune deposits utilized acetone-fixed frozen sections (4 μm) stained with Alexa Fluor-conjugated antibodies against IgA (1:200), C3 (1:500), IgM (1:400), and IgG (1:400). Mean fluorescence intensity was quantified using ImageJ across 20 glomeruli per mouse.

***Biochemical measurements***

Urinary albumin was quantified using a capture ELISA kit (Bethyl Laboratories), while creatinine levels were measured using commercial assay kits (BioAssay Systems for urine; Nanjing Jiancheng for serum). Proteinuria was expressed as albumin-to-creatinine ratio (ACR, mg/g). Blood urea nitrogen was assessed using a chemistry kit (BioAssay Systems). All assays were performed in duplicate according to manufacturer protocols.

# **Tables**

| **Gene name** | **Forward primer sequence** | **Reverse primer sequence** |
| --- | --- | --- |
| *C3* (mouse) | CCAGCTCCCCATTAGCTCTG | GCACTTGCCTCTTTAGGAAGTC |
| *Hc* (mouse) | GAACAAACCTACGTCATTTCAGC | GTCAACAGTGCCGCGTTTT |
| *Il10* (mouse) | GCTCTTACTGACTGGCATGAG | GCTCTTACTGACTGGCATGAG |
| *Il17f* (mouse) | TGCTACTGTTGATGTTGGGAC | AATGCCCTGGTTTTGGTTGAA |
| *Ccl2* (mouse) | TTAAAAACCTGGATCGGAACCAA | TTAAAAACCTGGATCGGAACCAA |
| *Cxcl10* (mouse) | CCAAGTGCTGCCGTCATTTTC | GGCTCGCAGGGATGATTTCAA |
| *Il1b* (mouse) | GCAACTGTTCCTGAACTCAACT | ATCTTTTGGGGTCCGTCAACT |
| *Il6* (mouse) | CCAAGAGGTGAGTGCTTCCC | CTGTTGTTCAGACTCTCTCCCT |
| *Tgfb1* (mouse) | CTCCCGTGGCTTCTAGTGC | GCCTTAGTTTGGACAGGATCTG |
| *Gapdh* (mouse) | AGGTCGGTGTGAACGGATTTG | TGTAGACCATGTAGTTGAGGTCA |

## Supplemental Table S1. RT-qPCR primer sequences.

# **Figures**

## Supplementary Figure S1. Validation of *C3* knock out in *C3*^-/-^ mice.

*C3* expression in liver tissues of WT and *C3*^-/-^ mice detected by RT-qPCR (left). Plasma C3 levels in WT and *C3*^-/-^ mice measured by ELISA (right). Data are presented as mean ± SEM; *p ≤ 0.05, **p ≤ 0.01, ***p ≤ 0.001.


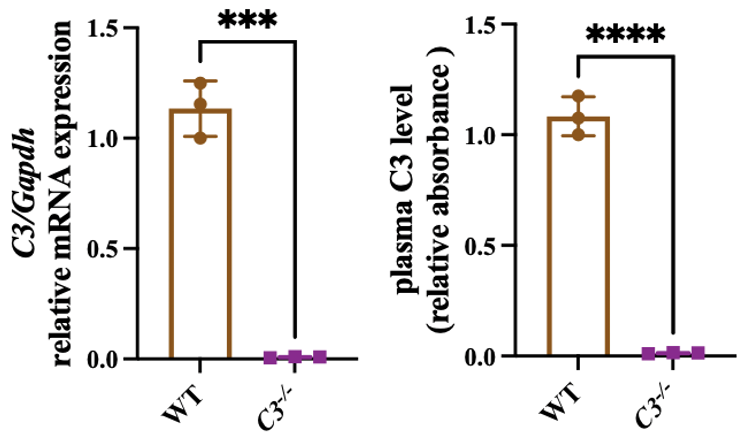


Supplementary Figure S2. Validation of recombinant mouse Cfh protein.
(a) Coomassie Brilliant Blue staining showed a single band corresponding to the expected molecular weight of mCfh, confirming high purity. (b) Western blot analysis using an anti-CFH antibody verified the identity of the recombinant protein. (c) In a hemolysis assay using sheep erythrocytes under conditions of alternative pathway activation, the addition of increasing concentrations of purified mCfh resulted in a dose-dependent inhibition of hemolysis, confirming its complement regulatory function. Data are presented as mean ± SEM; ****p ≤ 0.001.


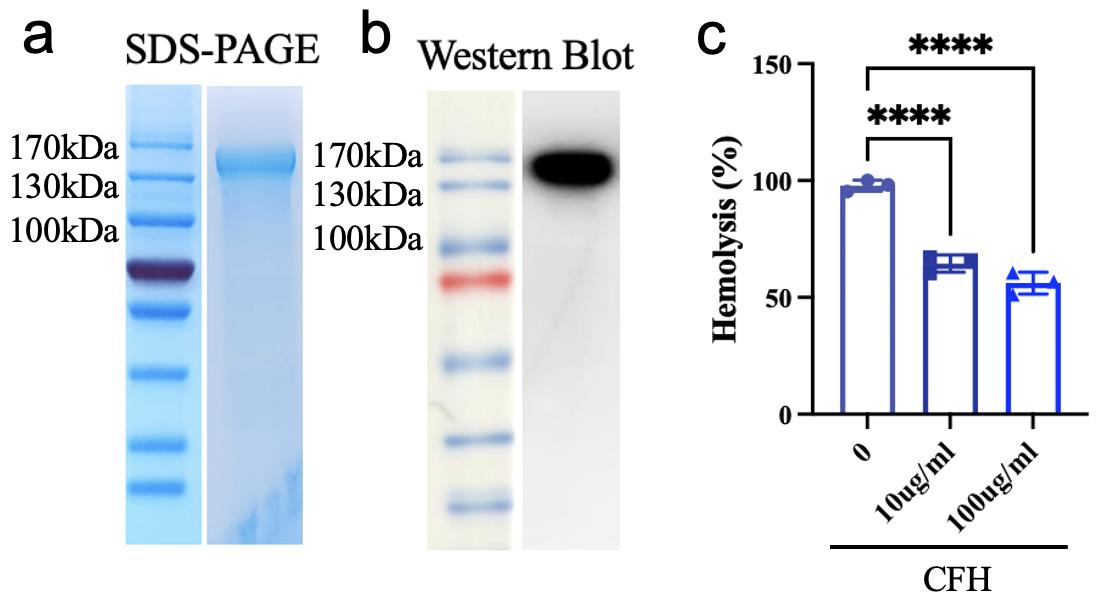


## Supplementary Figure S3. Detection of mCfh on mouse platelets.

Flow cytometric detection of complement factor H bound to mouse platelets with and without addition of mouse Cfh. *p ≤ 0.05, **p ≤ 0.01, ***p ≤ 0.001; ns, not significant (p > 0.05).


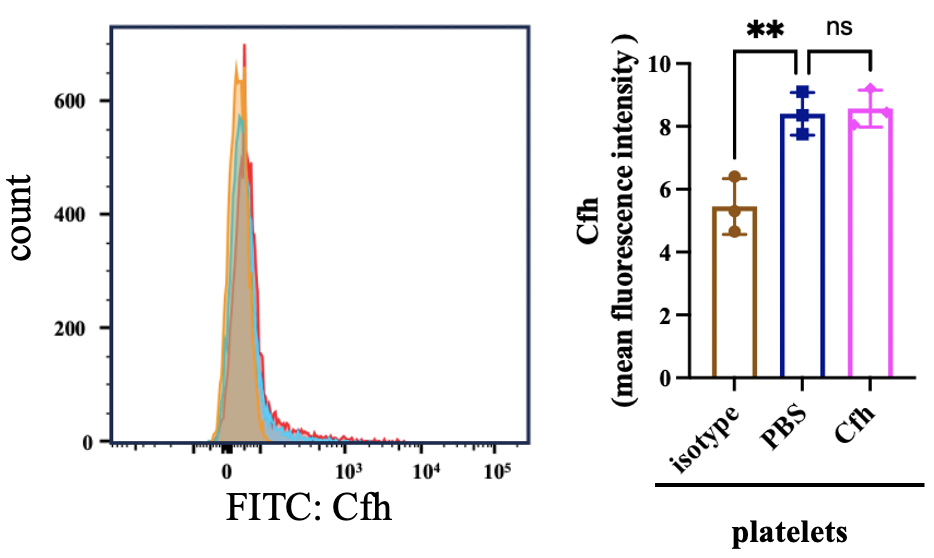


## Supplementary Figure S4. Detection of IgA containing immune complexes on human erythrocytes.

(a) Flow cytometric analysis of IgA containing immune complexes binding to human erythrocytes. (b) Flow cytometric analysis of IgA containing immune complexes binding to human erythrocytes with and without addition of human CFH. *p ≤ 0.05, **p ≤ 0.01, ***p ≤ 0.001; ns, not significant (p > 0.05).


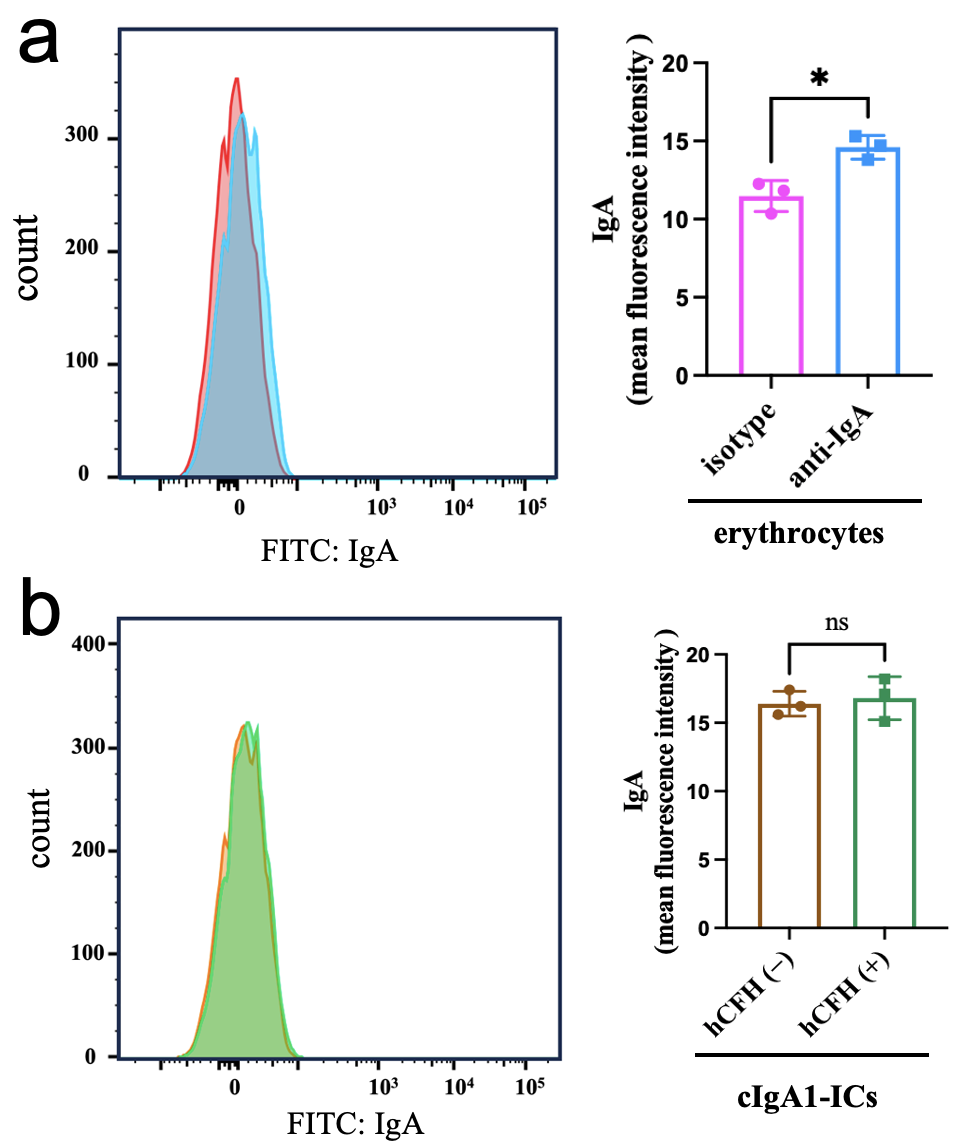


## Supplementary Figure S5. Plasma CFH levels in IgAN patients stratified by severity of proteinuria.

(a) No significant correlation was found between plasma CFH levels and 24-hour urinary total protein (UTP) in IgAN patients. ns, not significant. (b) Plasma CFH levels did not significantly differ between IgAN patients with or without nephrotic range proteinuria. *p ≤ 0.05, **p ≤ 0.01, ***p ≤ 0.001; ns, not significant (p > 0.05).


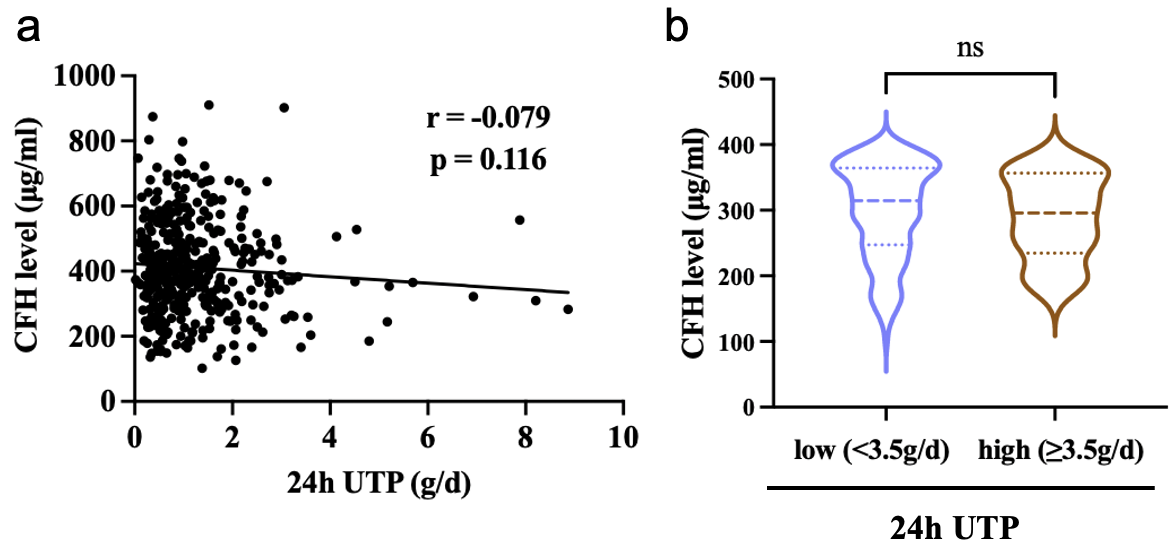

Supplement: Supplementary file 1 — Supplementary Material 1. [file 43556_2026_463_MOESM1_ESM.docx]
